# A Machine Learning Approach to the Interpretation of Cardiopulmonary Exercise Tests: Development and Validation

**DOI:** 10.1155/2021/5516248

**Published:** 2021-05-31

**Authors:** Or Inbar, Omri Inbar, Ronen Reuveny, Michael J. Segel, Hayit Greenspan, Mickey Scheinowitz

**Affiliations:** ^1^Department of Biomedical Engineering, Tel-Aviv University, Israel; ^2^The Edmond and Lily Safra Children's Hospital, Sheba Medical Center, Tel Hashomer, Israel; ^3^Pulmonary Institute, Sheba Medical Center Tel-Hashomer, Israel; ^4^Sackler School of Medicine, Tel-Aviv University, Tel-Aviv, Israel

## Abstract

**Objective:**

At present, there is no consensus on the best strategy for interpreting the cardiopulmonary exercise test's (CPET) results. This study is aimed at assessing the potential of using computer-aided algorithms to evaluate CPET data for identifying chronic heart failure (CHF) and chronic obstructive pulmonary disease (COPD).

**Methods:**

Data from 234 CPET files from the Pulmonary Institute, at Sheba Medical Center, and the Givat-Washington College, both in Israel, were selected for this study. The selected CPET files included patients with confirmed primary CHF (*n* = 73), COPD (*n* = 75), and healthy subjects (*n* = 86). Of the 234 CPETs, 150 (50 in each group) tests were used for the support vector machine (SVM) learning stage, and the remaining 84 tests were used for the model validation. The performance of the SVM interpretive module was assessed by comparing its interpretation output with the conventional clinical diagnosis using distribution analysis.

**Results:**

The disease classification results show that the overall predictive power of the proposed interpretive model ranged from 96% to 100%, indicating very high predictive power. Furthermore, the sensitivity, specificity, and overall precision of the proposed interpretive module were 99%, 99%, and 99%, respectively.

**Conclusions:**

The proposed new computer-aided CPET interpretive module was found to be highly sensitive and specific in classifying patients with CHF or COPD, or healthy. Comparable modules may well be applied to additional and larger populations (pathologies and exercise limitations), thereby making this tool powerful and clinically applicable.

## 1. Introduction

In the last three decades, clinical exercise testing, in general, and cardiopulmonary exercise testing, in particular, have emerged as an increasingly important tool for patient evaluation in clinical medicine due to a growing awareness of the limitations of traditional resting cardiopulmonary measurements [1]. As noted in the American Heart Association (AHA) Scientific Statement of 2010 [2] “CPET provides a wide array of unique and clinically useful incremental information that heretofore has been poorly understood and underutilized by the practicing clinician.” Other authors [3] have pointed out that the data generated from CPET are one of the most challenging sets of results to interpret. They also claim that the resources available to help physicians in the interpretation of CPET results are limited. They state that “…although the American Thoracic Society (ATS)/American College of Chest Physicians (ACCP) statement [4] is comprehensive, it must be approached with “zeal“ in order not to be overwhelmed” [3].

Almost all published CPET interpretive strategies are performed manually following expert-based guidelines [4–8]. These interpretation strategies, including flow charts and tables, are cumbersome, complicated, time-consuming, force dichotomous decision making, and partly subjective. They require extensive knowledge and understanding of the meaning and implications of the many CPET variables. As such, potential exists for inconsistent and sometimes inaccurate interpretation of CPET results [9, 10]. This may be at the core of why such a valued and noninvasive procedure (CPET) is underused [2, 11].

At present, there is no consensus on any reported interpretation strategy for CPET test results [4, 10]. In a recent study, Chacey et al. [12] carried out a retrospective review of 77 randomly chosen CPET files to determine the presence of inconsistencies in CPET interpretation from the guidelines issued by ATS/ACCP [4]. They reported that 78% of interpreted CPET studies contained at least one inconsistency. Furthermore, except for Schmid et al. [10], none of the available algorithms were clinically validated [4, 10, 13].

The present study is aimed at assessing the potential of using computer-aided algorithms to evaluate CPET data to identify individuals suffering from chronic heart failure (CHF) or chronic obstructive pulmonary disease (COPD) or are healthy.

In trying to achieve the above goal, we have used classification modules using machine learning algorithms (MLA) such as the support vector machine (SVM). MLAs are increasingly being used in clinical research [14, 15]. Their modeling flexibility makes them valuable tools, especially to describe complex relationships between the outcome and the predictors. Furthermore, in contrast to the standard statistical methods, they do not make any parametric assumptions, which may be potentially advantageous in small studies where the assumptions of classical methods often do not hold. SVM models are used for combining biomarkers through machine learning algorithms in which numerous variables are integrated by a computer program that is first taught to associate one specific clinical value with a combination of dataset [16]. The learned algorithm is then applied to new datasets. It is a model-free method that provides efficient solutions to classification problems without any assumptions regarding the distribution and interdependency of the data. Therefore, it is well suited to be used in studies encompassing multiple factors with minor effects, limited sample sizes, and limited knowledge of underlying biological relationships among attributes [17, 18]. Unsupervised clustering and supervised categorization schemes employed by the SVM facilitate the analysis of large amounts of high-dimensional feature vectors (entailing, in this case, a large set of patient descriptors) [19]. Using clustering techniques enables the automated definition of homogeneous subgroups within the data. In supervised SVM classification, one can learn to model a particular category of patients or discriminate between pathologies and their severity [20].

We hypothesized that a supervised computerized learning algorithm, when given appropriate data from CPET studies, would achieve an acceptable agreement for a major or primary diagnosis with the diagnosis made by conventional manual interpretation.

## 2. Methods

### 2.1. Participants

This study used 234 retrospective CPET files (177 men and 57 women), of which 148 were previously diagnosed as having either primary illness, CHF (*n* = 73) or COPD (*n* = 75), or were considered healthy (*n* = 86). The CHF and the COPD patients (*n* = 148) were clinically diagnosed and treated in the cardiology or the pulmonary departments at the Sheba Medical Center in Ramat-Gan. It should be pointed out that some of the studied patients presented with the coexistence of CHF and COPD, and their final group assignment was based on the most prominent clinical findings and symptoms (primary or secondary). The CPET files of the healthy participants were obtained through the CPET database at the exercise physiology laboratory of the Givat-Washington College in Israel. The equipment and all tests' protocols were the same in the two laboratories. The primary criteria for inclusion in the study cohort were valid and confirmed diagnosis of either CHF, COPD, or healthy, technically sound CPET, technically good pulmonary function Test (PFT), maximal effort or symptom-limited CPET tests (respiratory exchange ratio (RER) ≥ 1.00; test duration ≥ 6 min) and age ≥ 25 years old). Healthy normal subjects were older than 25 years, have no history of chronic diseases, have normal cardiorespiratory fitness, and are otherwise in good health. Senior cardiologists and pulmonologists made all clinical diagnoses. The conventional clinical diagnoses of the CHF and COPD patients were made according to the ATS and the American Heart Association (AHA) respective guidelines [21–23] and included some or all of the following procedures; for COPD: spirometry, bronchodilator reversibility, blood tests, chest X-ray or CT scan, sputum examination, and electrocardiogram (ECG) and for CHF: blood tests, chest X-ray, ECG, echocardiogram, stress test, cardiac CT scan, MRI, and, coronary angiogram. CPETs were not included in the conventional clinical diagnostic procedures.

This study was conducted following the amended Declaration of Helsinki. The Institutional Review Board (IRB) of the Sheba Medical Center approved the protocol (No. 1730-14-SMC). Informed consent was not required due to the observational and retrospective nature of the study design.

**Figure 1:**
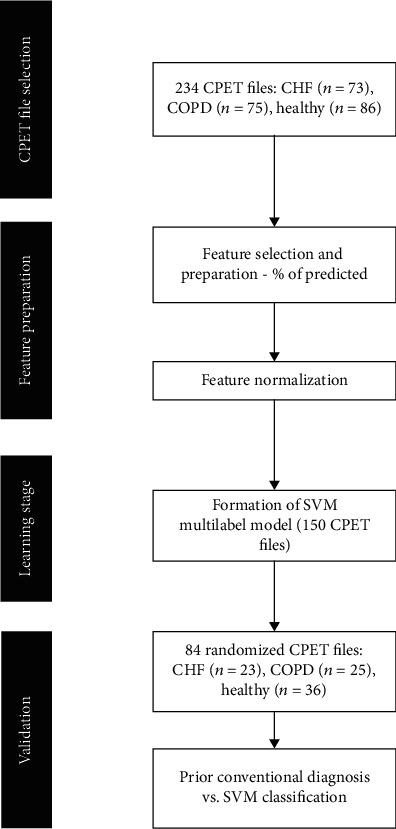
Schema of the study's design.

A flow chart of the study design is shown in Figure 1.

### 2.2. The Cardiopulmonary Exercise Test (CPET)

Before performing the CPET, all study participants completed a pulmonary function test, according to the ATS guidelines [23]. The participants were seated on a cycle ergometer (Ergoselect 1200, Germany). Following a 3 min rest period and 3 min of unloaded pedaling, an incremental symptom-limited maximal exercise test was performed. Expired O_2_ and CO_2_ gases and the airflow rate were measured breath-by-breath through a facemask connected to a metabolic cart (all from COSMED, Italy). Gas analyzers (O_2_ and CO_2_) were calibrated before each test. The airflow sensor was calibrated daily. The exercise protocols were designed to ensure that subjects reached volitional exhaustion within 8-12 minutes of incremental exercise. Work rate increments ranged from 5 to 25 watts.min-1.

Before entering the CPET data into the selected SVM learning and the respective validation processes, maximal and submaximal values of each CPET file were obtained using conventional algorithms embedded in the metabolic cart (COSMED, Italy).

Then, the relations of those measured values to their corresponding normal (predicted) values were calculated (% of predicted). The predicted normal values were based on Inbar et al. [24] and Wasserman et al. [6] CPET's reference values. The use of % predicted values as input data for the SVM assured unbiased comparisons of the various physiological attributes (peak and submaximal) among wide-ranging test protocols, ergometers, and populations of varied physical, physiological, and pathological characteristics.

### 2.3. Normalizing Ranges of % of Predicted Values (80%-100%)

During CPET, assorted physiological variables are measured with their widely spread respective normal peak values [6, 24]. Table s-1 in the supplementary materials presents an example of normal peak values of a COPD patient and a healthy one, both at age 62 years, and their respective % of predicted ranges and the resulting limits of their % predicted values for two selected CPET attributes (HR and VE). As shown in table s-1, there are widespread spans in the % of predicted values among the displayed CPET attributes (see table s-1, column 7). As shown, one could have a significantly lower than predicted normal peak HR (i.e., 114 beats/min—see column 2), resulting in a 71% of the predicted normal (predicted normal range is 96%-104%; see table s-1, column 7). Simultaneously, a normal healthy peak VE value of 40 L/min will also result in 71% of predicted normal (see table s-1, Column 2) while the predicted normal range is 71%-129% - see table s-1, column 7).

Such cases could hamper the SVM's learning phase and hinder the optimal SVM classification performance [25].

To overcome the above problem and standardize the ranges of the CPET predicted normal limits, we rescaled the original boundaries of all expected normal ranges into equal limits of 80% and 100% of predicted normal (commonly used in medical sciences). It was done by applying a linear regression equation for each CPET variable using three points: the *lower* limit of the predicted normal range was set as 80% of normal, the *average* of the predicted normal range was set as 90% of the normal, and the *upper limit* of the predicted normal range was set as 100% of the normal.

Feature scaling is mapping the feature values of a dataset into the same range and is crucial for machine learning algorithms such as the SVM [25]. Training an SVM classifier includes deciding on a boundary between classes. This boundary is known to have the maximum distance from the nearest point on each data class and differs for nonscaled and scaled cases. Also, the linear scaling of the input data in our study was done to avoid attributes with greater numeric ranges that could dominate those with smaller numeric ranges [25].

Table s-2 in the Supplementary Materials presents comparisons between nonnormalized and normalized CPET values (% predicted) as the input features for the multilabel SVM interpretive model design (from here on will be designated as % of predicted). Table s-2 demonstrates the advantage of using normalized rather than nonnormalized CPET values as input features for the SVM model design.

Following the feature preparation, the SVM learning stage was employed. To explore the high-dimensional space of CPET parameters towards creating the novel rule, discovery correlations, and criteria for disease characterization, we used a linear SVM (multiclass) machine learning tool. The evaluation of the SVM classification results was based upon the SVM probability estimates.

We used SVM procedures to identify (classify) three distinct populations: two highly prevalent chronic diseases, CHF and COPD, and healthy normal subjects (Healthy).

### 2.4. The SVM Algorithms

SVM is a supervised machine learning technique that is widely used in pattern recognition and classification problems. It includes a set of supervised learning methods developed in the 1990s [17, 20] and is used to solve classification and regression problems. SVM is one of the most popular techniques for supervised classification [26], built on the structural risk minimization (SRM) induction principle, and has found success in a variety of applications [27]. However, the success of many applications using the SVM critically depends on the initial manual choice of features. As indicated above and since this study deals with populations with varied pathophysiological responses during an incremental exercise challenge (due to gender differences, age, weight, height, and physical condition), we used maximal and submaximal CPET values related to respective/relevant normal CPET values (% of predicted values) for the SVM input data (see further elaboration on this issue above).

The SVM model implementations in this study were executed using the Library for Support Vector Machines (LIBSVM) toolbox in MATLAB R2013b [28].

### 2.5. The SVM Learning Stage

For the SVM learning stage, 150 retrospectively diagnosed individuals with CHF (*N* = 50) and COPD (*N* = 50), as well as healthy participants (*N* = 50) were randomly selected. Patients with varying degrees of disease severity (mild, moderate, and severe) and varying fitness levels (healthy) were included in this stage.

For this stage, we used the Library for Support Vector Machines (LIBSVM) linear multilabel classifier as a learning tool [28, 29] for the three study groups (CHF, COPD, and healthy patients). The SVM multilabel classification model was created based on the input of all CPET parameters (% of predicted).

### 2.6. The SVM Model Cross-Validation

To evaluate the consistency of the estimates from the newly created SVM model, 4-fold cross-validation procedures were performed on the learning dataset. In each cross-validation stage, the learning dataset was split into the training and validation datasets. This cross-validation process was repeated numerous times (iterations) (see Table 1), allowing each subset to serve once as the test dataset.

### 2.7. Validation of the Classification Stage

For this stage, the remaining 84 CPET files were added: 23 patients with CHF, 25 with COPD, and 36 healthy participants. Patients with varying degrees of disease severity (mild, moderate, and severe) and varying fitness levels (healthy) were included in this stage. The SVM disease classification (CHF, COPD, or healthy) was based on the SVM probability estimation [30]. A given disease was classified concurring with its highest SVM probability estimate. The SVM classification outcomes (probability estimation) were then compared with the prior official clinical diagnosis.

As indicated above, the validation group included several patients with coexisting respiratory/cardiac illnesses (and in some cases other, more minor diseases). Such a cohort provided more representative patients' samples and consequently a more sensitive assessment of the actual diagnostic accuracy of this algorithm.

### 2.8. Statistical Analyses

Discrete values (participants' physical characteristics and CPET peak and submaximal values) were calculated and are presented as means ± standard deviation (SD). Comparisons among groups were performed by one-way analysis of variance (ANOVA) (see s-Table 3 and s-Table 4).

The result of the SVM disease classification for each CPET test was compared with its corresponding original clinical diagnosis and considered true positive (TP), false positive (FP), true negative (TN), or false negative (FN). Sensitivity, specificity, accuracy, and overall precision were calculated based on the following formulas:

(1) $$\begin{array}{c} \text{Sensitivity}=\frac{\text{TP}}{\text{TP}+\text{FN}}, \end{array}$$

(2) $$\begin{array}{c} \text{Sensitivity}=\frac{\text{TN}}{\text{TN}+\text{FP}} \end{array}$$

(3) $$\begin{array}{c} \text{Accuracy}=\frac{\left(\text{TP}+\text{TN}\right)}{\text{TP}+\text{FP}+\text{FN}+\text{TN}}, \end{array}$$

(4) $$\begin{array}{c} \text{Precision}=\frac{\text{TP}}{\text{TP}+\text{FP}}. \end{array}$$

TP, FP, TN, and FN represent the number of true positives, false positives, true negatives, and false negatives. A *p* value of ≤ 0.05 was considered statistically significant.

## 3. Results

### 3.1. Participants

The physical characteristics of all study participants (197 males and 37 females) of both the learning and the validation stages, by groups, are summarized in Table 2.

### 3.2. CPET Results

**Figure 2:**
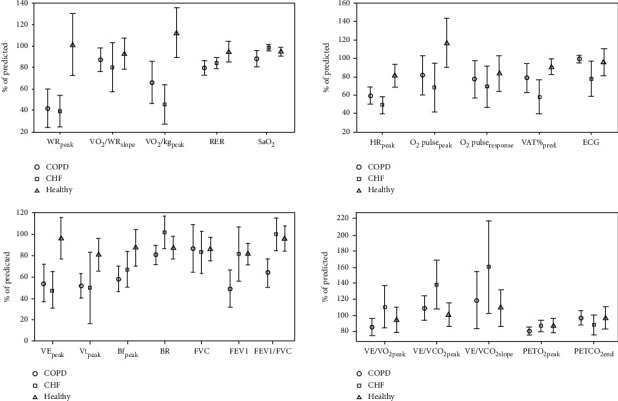
The distribution of CPET results (normalized % of predicted) of all CPET variables of the three study groups (mean ± SD)—validation stage.

CPET results, both in absolute and relative (normalized % of predicted) values of all participants by stages (learning and validation) are presented in s-Table 3 and s-Table 4. Focusing on s-Table 4 in the supplementary materials (validation stage), significant differences were observed between the CPET values (normalized % of predicted) of the two patients' groups (CHF and COPD) in half of the CPET attributes (peak VO_2_/kg, peak HR, ECG, VAT, peak SaO_2_, peak BR, peak VE/VO_2_, peak VE/VCO_2_, VE/VCO_2_ slope, FEV1, and FEV1/FVC) (for respective abbreviations see denotes of s-Table 3). CPET variables differed significantly among the three studied groups (see Table 4 in the supplementary materials and Figure 2 in the text). Therefore, one may argue that, with multiple variables showing significant differences among the three studied groups, it should not be too difficult and time-consuming to discriminate between the three groups, even manually. Nevertheless, when closely examining Figure 2, it is apparent that there is a substantial overlap in the individual data points in most of the variables measured in the three studied groups. Such overlap could, at least partially, explain the complexity and inconsistency of interpreting individual CPET results. It should be accentuated that the presented dichotomized diagnoses (CHF, COPD, and healthy), reflects, in those demonstrated coexisting pathologies, the primary pathology only (highest probability estimates (%)).

### 3.3. The Cross-Validation

Table 1 summarizes the results of the cross-validation processes estimating how accurate the SVM-created predictive multilabel model will perform in practice.

In this stage (learning), repeated random subsampling and leave-one-out cross-validation procedures were carried out on the training dataset. Repeated random subsampling cross-validation is a method that splits the dataset into training and validation data. In the present study, we used three splits of cross-validation. The first splits included 80% of the sample files for the model training and 20% of the sample files for the model validation. In the second and third splits, we used 70% for training and 30% for validation and 50% for training, and 50% for validation. Leave-one-out is a particular case of repeated random sub-sampling cross-validation where the validation dataset is 1. The results show a significant separation (very high SVM probability estimates) between the three study populations and a very high similarity within each group (low SDs). The above data revealed excellent learning performance and paved the way for the disease classification validation stage.

### 3.4. The SVM Disease Classification Validation

Tables 3–5 present the various outcomes of the validation stages.

Table 3 presents the summary of groups' means (±SD) of the individual SVM disease classification outcome (probability estimation (%)).

Nonetheless, the level of the probability estimates varied widely within each group, signifying clinical heterogeneity regarding disease severity. The inclusion of participants with varying disease severity and fitness levels (peak VO_2_/kg) reinforces the utilization of the proposed SVM classification models for patients with a wide range of disease severity and fitness levels.

Table 4 presents the confusion matrix of the SVM disease identification model and creates the basis for quantifying the performance of the SVM disease classification (Table 5).

Table 5 demonstrates the performance quantification of the SVM disease identification model.

The SVM multilabel model's sensitivity, specificity, accuracy, and precision for classifying the three studied groups are very high (Table 5). The disease classification results show that the overall predictive power of the model ranged from 96% to 100%, indicating very high predictive power.

## 4. Discussion

The goal of the current study was to develop and validate a computer-aided algorithm for automatically assessing CPET test results, thereby classifying three distinct groups of patients, clinically diagnosed as having CHF, COPD, or being healthy, by using machine learning techniques (SVM).

In this study, we show that by uniquely converting CPET raw data of clinically/manually diagnosed CHF, COPD, and healthy patients (normalized % predicted values), and transmitting them through a machine learning process, we can discriminate between individuals suffering from CHF, COPD, or, are genuinely healthy, with very high accuracy. Therefore, the study's hypothesis was confirmed.

The proposed module combines two novel approaches for the interpretation process of CPET results; the first one was the use of supervised machine learning techniques (SVM), and the second one was the use of normalized percent of predicted normal (% predicted), rather than absolute CPET values. By doing so, it is possible to apply the proposed interpretive model to individuals with heterogeneous clinical, anthropometric, and demographic characteristics.

As shown in Figure 2, in all but four CPET features (WR, VO_2_/kg, HR, and VE), the individual data points of the corresponding variables widely overlap among the three study groups. It makes manual interpretation highly complex, confusing, and to a certain extent, subjective. We, therefore, sought to demonstrate that by using machine learning-based analysis of all CPET data, it would be possible to reliably distinguish between COPD, CHF, and healthy participants, irrespective of their comorbidities, disease severity, age, gender, and fitness level.

The results demonstrate that using SVM-based learning and prediction approaches revealed strong agreement with common clinical disease diagnosis, made by expert cardiologists and pulmonologists (sensitivity of 99%, specificity 99%, and overall precision of 99%) (see Table 5).

The successful use of this algorithm in combining pulmonary function test (PFT) and CPET features (attributes) is, to the best of our knowledge, the only reported effort to combine such input features (% of predicted normal) for computerized diagnostic purposes.

So far, only one study has attempted to validate some CPET interpretive strategies [10] systematically. In this study, a newly proposed manual interpretive strategy was compared with a more conventional alternative [6] for evaluating CPET results. Although the consistency of the proposed interpretation method was relatively high (82%), it suffers from the previously mentioned disadvantages of most manually performed CPET interpretation schemes [12]. Moreover, in Schmid et al.'s study [10], blood gas analyses were performed during CPET, which is rarely used during routine CPETs.

Furthermore, in the single published attempt to computerize CPET interpretation, Ross and Corry [9] used absolute rather than relative (% of predicted) CPET values. Using “crude” CPET values refutes the use of such an interpretation strategy in heterogeneous populations (i.e., gender, age, pathologies, and fitness level). Also, the above computer-aided interpretation algorithm was never validated.

As it has in many sciences and other complex endeavors, interpretation software will undoubtedly become helpful in facilitating medical diagnoses and implementing appropriate therapies. A recent attempt to employ the machine learning (ML) technique in identifying cause/s for the unexplained reduced exercise capacity in lung transplant recipients using CPET data, and some additional external attributes (primarily subjective) showed promising results [31].

The present endeavor represents a novel and substantial addition in medical interpretive software to assist inpatient care.

The use of machine learning technology combined with a relative (% of predicted) rather than absolute input features opens up promising prospects for additional efforts to develop computer-aided modules to classify other pathologies, causes, and severity of exercise intolerance.

### 4.1. Study Limitations

The main shortcoming of the current study is the inclusion of only three sample populations (COPD, CHF, and healthy). As noted, this was a proof-of-principle study, which will lead to broader applications of the SVM methods in future work.

Also, the accuracy and precision of using such analysis (SVM) will be limited by the quality of the CPET raw data. CPET data could be affected by device limitations (sensors' accuracy) and the quantification process. Quality problems in the CPET data could also arise from the dependence on technical limitations of the currently available devices, including the one used here.

### 4.2. Conclusions

In this research work, the SVM classification process was used to identify, based on CPET data, three distinct sample populations, CHF, COPD, and healthy. Comparisons of SVM prediction outcomes with the respective conventional clinical diagnoses were made based on classifying each study participants' performance accuracy. Our results demonstrate that the discriminative performance of the SVM model matched perfectly with the official conventional clinical diagnosis, with the latter involving various costly and time-consuming clinical and lab procedures. Using such computer-aided techniques will reduce complexity, increase objectivity, and economize on CPET interpretation in clinical settings.

To our knowledge, this is the first study demonstrating that an automated classification approach using SVM can be used successfully to detect common chronic diseases with a single, short, noninvasive, and relatively inexpensive laboratory test such as CPET.

It should be pointed out that the presented report is the first part (being proof-of-principle one) of a larger project aimed at using the SVM technique for classifying several additional clinical conditions as well as types and severity of exercise limitations.

## Figures and Tables

**Table 1:** Outcomes (probability estimates (%)) of the SVM multilabel crossvalidation procedure of the learning stage.

Conventional clinical diagnosis	Model cross-validation		SVM classification (%)		
	Sample's splits	No. of iterations	CHF	COPD	Healthy
CHF	Leave-one-out	150	96 ± 5	3 ± 4	2 ± 2
COPD			3 ± 6	95 ± 7	1 ± 2
Healthy			2 ± 2	2 ± 2	96 ± 4
CHF	80% (training) 20% (validation)	300	95 ± 4	3 ± 3	2 ± 2
COPD			6 ± 11	93 ± 11	1 ± 1
Healthy			1 ± 1	2 ± 2	97 ± 3
CHF	70% (training) 30% (validation)	675	95 ± 4	3 ± 2	2 ± 2
COPD			6 ± 11	93 ± 11	2 ± 2
Healthy			2 ± 2	2 ± 2	96 ± 4
CHF	50% (training) 50% (validation)	1875	93 ± 5	4 ± 3	3 ± 3
COPD			6 ± 9	92 ± 9	2 ± 2
Healthy			3 ± 3	3 ± 3	94 ± 5

Data presented as mean ± SD. CHF: chronic heart failure; COPD: chronic obstructive pulmonary disease; Healthy: healthy normal participants; SVM: support vector machine, SD: standard deviation. Bold numbers denote mean ± SD (%) probability estimates of the respective group.

**Table 2:** Physical characteristics of the study participants.

Study stage	Variable	CHF	COPD	Healthy
Learning stage *N* = 150 (113 M, 37 F)	Age (yr)	52.2 ± 13.3	64.4 ± 10.2	45.7 ± 9.3
	Height (cm)	172.3 ± 6.2	169.0 ± 6.7	173.0 ± 4.5
	Weight (kg)	79.4 ± 11.7	70.7 ± 13.3	76.6 ± 5.6

Validation stage *N* = 84 (64 M, 20 F)	Age (yr)	53.7 ± 13.6	66.8 ± 7.6	37.8 ± 13.8
	Height (cm)	172.2 ± 6.9	166.5 ± 6.3	168.9 ± 9.1
	Weight (kg)	81.2 ± 14.9	74.7 ± 14.4	65.1 ± 14.1

Data are presented as mean ± SD. CHF: chronic heart failure; COPD: chronic obstructive pulmonary disease; Healthy: healthy normal participants; M: males; F: females.

**Table 3:** Summary of the individual probability estimates (%) of the SVM disease classification model.

Conventional clinical diagnosis	Patients' group	SVM probability estimation (%)			
		Mean	SD	Min	Max
CHF	CHF	**92.4**	**10.4**	**57.0**	**100.0**
	COPD	4.2	6.1	0.0	27.0
	Healthy	3.3	5.7	0.0	19.0

COPD	CHF	10.6	13.2	0.0	47.0
	COPD	**79.6**	**16.9**	**45.0**	**100.0**
	Healthy	8.7	13.1	0.0	46.0

Healthy	CHF	5.6	7.8	0.0	36.0
	COPD	8.5	11.7	0.0	43.0
	Healthy	**85.4**	**17.1**	**43.0**	**100.0**

CHF: chronic heart failure; COPD: chronic obstructive pulmonary disease; Healthy: healthy normal participants; SVM: support vector machine; SD: standard deviation; Min: minimum; Max: maximum. Bold numbers denote average probability estimates of the respective group.

**Table 4:** Performance summary of the SVM classification model.

Group	TP	FN	FP	TN
CHF	23	0	1	60
COPD	24	1	0	59
Healthy	36	0	0	48

CHF: chronic heart failure; COPD: chronic obstructive pulmonary disease; Healthy: healthy normal participants; TP: true positive; FN: false negative; FP: false positive; TN: true negative.

**Table 5:** Performance quantification of the SVM disease identification model (%).

Group	Sn (%)	Sp (%)	Acc (%)	Pr (%)
CHF	100	98	99	96
COPD	96	100	99	100
Healthy	100	100	100	100
Mean	**99**	**99**	**99**	**99**

CHF: chronic heart failure; COPD: chronic obstructive pulmonary disease; Healthy: healthy normal participants; Sn: sensitivity; Sp: specificity; Acc: accuracy; Pr: precision.

## Data Availability

The data used to support the findings of this study are included within the supplementary information files. Additional data could be obtained from Or Inbar at orinbar10@gmail.com.

## Abbreviations

ACCP: American College of Clinical Pharmacy
ATS: American Thoracic Society
CHF: Chronic heart failure
COPD: Chronic obstructive pulmonary disease
CPET: Cardiopulmonary exercise testing
ECG: Electrocardiogram
SD: Standard deviation
ML: Machine learning
SVM: Support vector machine.

## Supplementary Materials

Supplementary 1 s-Table 1: an example of measured normal peak HR and VE and their respective normal ranges, and the resulting limits of their % predicted values for a 62-year-old male COPD patient and a 62-year-old healthy male.

Supplementary 2 s-Table 2. Comparison between nonnormalized and normalized CPET attributes (% predicted) as features for the multilabel SVM interpretive model design.

Supplementary 3 s-Table 3. Comparisons of CPET results (actual and % of predicted) among the three studied groups—the learning stage.

Supplementary 4 s-Table 4. Comparisons of CPET results (actual and % of predicted) among the three studied groups—the validation stage.
