# Supplementary material for: A Machine Learning Approach to the Interpretation of Cardiopulmonary Exercise Tests: Development and Validation
Source: Pulm Med. 2021 May 31;2021:5516248. doi: 10.1155/2021/5516248 (PMC8188599; doi:10.1155/2021/5516248)
Supplement: Supplementary 1 — s-Table 1: an example of measured normal peak HR and VE and their respective normal ranges, and the resulting limits of their % predicted values for a 62-year-old male COPD patient and a 62-year-old healthy male. [file 5516248.f1.docx]

**s-Table 1.** An example of measured normal peak HR and VE and their respective normal ranges, and the resulting limits of their % predicted values for a 62-year-old male COPD patient and a 62 -year-old healthy male.

| **Parameter** | **Measured value** | **% of predicted** | **Predicted normal value** | **SEE** | **Predicted normal range** | **% of predicted normal range** |
| --- | --- | --- | --- | --- | --- | --- |
| **HR, [beat/min]** | **114** [beat/min] – (**COPD patient**) | **71%** | 161.5 | 6.5 | 155-168 | 96%-100% |
| **VE,**  **[L/min]** | **40** [L/min] – **(healthy subject**) | **71%** | 56 | 16 | 40-172 | 71%-129% |

HR = heart rate; VE = minute ventilation; Predicted normal value = predicted mean peak value; SEE = Standard Error of Estimate; Predicted normal range = predicted normal value ± SEE; % of predicted normal range = Predicted normal range, in percentage.
