# Supplementary material for: A Machine Learning Approach to the Interpretation of Cardiopulmonary Exercise Tests: Development and Validation
Source: Pulm Med. 2021 May 31;2021:5516248. doi: 10.1155/2021/5516248 (PMC8188599; doi:10.1155/2021/5516248)
Supplement: Supplementary 2 — s-Table 2. Comparison between nonnormalized and normalized CPET attributes (% predicted) as features for the multilabel SVM interpretive model design. [file 5516248.f2.docx]

**s-Table 2.** Comparison between non-normalized and normalized CPET attributes (% predicted) as features for the multi-label SVM interpretive model design

| Population | % predicted | Sn (%) | | Sp (%) | | Acc (%) | | Pr (%) |
| --- | --- | --- | --- | --- | --- | --- | --- | --- |
| CHF | **Normalized** | **100** | **98** | | **99** | | **96** | |
|  | **Non-Normalized** | 100 | 92 | | 94 | | 81 | |
| COPD | **Normalized** | **96** | **100** | | **99** | | **100** | |
|  | **Non-Normalized** | 71 | 100 | | 91 | | 100 | |
| Healthy | **Normalized** | **100** | **100** | | **100** | | **100** | |
|  | **Non-Normalized** | 95 | 92 | | 93 | | 89 | |

CHF = Chronic Heart Failure; COPD = Chronic Obstructive Pulmonary Disease; Healthy = healthy normal participants; % predicted = percent of predicted normal value (input features for the SVM model); Sn = Sensitivity; Sp = Specificity; Acc = Accuracy; Pr = Precision.
