# Supplementary material for: A Machine Learning Approach to the Interpretation of Cardiopulmonary Exercise Tests: Development and Validation
Source: Pulm Med. 2021 May 31;2021:5516248. doi: 10.1155/2021/5516248 (PMC8188599; doi:10.1155/2021/5516248)
Supplement: Supplementary 3 — s-Table 3. Comparisons of CPET results (actual and % of predicted) among the three studied groups—the learning stage. [file 5516248.f3.docx]

| **Variables** | **CHF (n=50)** | | **COPD (n=50)** | | **Healthy (n=50)** | |
| --- | --- | --- | --- | --- | --- | --- |
|  | **Measured^a^** | **% of pred.^d^** | **Measured^b^** | **% of pred.^e^** | **Measured** **^c^** | **% of pred.^f^** |
| **Time [min]** | 9.3±2.4**^b^** | 90.4±9.5 | 10.9±2.4**^a^** | 95.7±16.5 | 9.9±1.8 | 91.8±8.1 |
| **Peak WR [watt]** | 83.5±35.0**^bc^** | 43.8±13.0**^f^** | 57.7±19.3**^ac^** | 42.6±10.8**^f^** | 180.2±27.1**^ab^** | 90.7±6.8**^de^** |
| **Peak VO_2_ [l/min]** | 1.1±0.3**^c^** | 45.2±13.1**^ef^** | 1.2±0.4**^c^** | 60.0±15.7**^df^** | 2.5±0.2**^ab^** | 92.2±9.8**^de^** |
| **Peak VCO_2_ [l/min]** | 1.1±0.4**^c^** | 35.8±10.6**^ef^** | 1.1±0.5**^c^** | 43.5±15.0**^df^** | 3.1±0.5**^ab^** | 88.9±7.1**^de^** |
| **RER** | 1.08±0.06**^c^** | 85.0±4.3**^f^** | 1.08±0.07**^c^** | 85.0±4.8**^f^** | 1.19±0.09**^ab^** | 92.7±6.6**^de^** |
| **Peak VO_2_/kg [ml/kg/min]** | 13.3±4.2**^bc^** | 42.2±9.7**^ef^** | 16.9±3.7**^ac^** | 64.3±10.1**^df^** | 32.4±4.7**^ab^** | 91.0±6.5**^de^** |
| **VO_2_/WR slope** | 7.6±1.0**^bc^** | 71.1±8.0**^ef^** | 10.4±1.1**^a^** | 90.4±6.0**^d^** | 10.2±1.2**^a^** | 89.2±7.4**^d^** |
| **Peak HR [beat/min]** | 103.7±13.1**^bc^** | 50.8±6.1**^ef^** | 114.0±14.3**^ac^** | 58.8±6.4**^df^** | 175.1±10.2**^ab^** | 91.1±7.0**^de^** |
| **Peak O_2_Pulse [(ml/kg/beat)x100]** | 12.1±1.9**^bc^** | 65.9±8.4**^ef^** | 13.8±2.5**^ac^** | 80.4±11.2**^df^** | 19.0±2.1**^ab^** | 93.5±6.7**^de^** |
| **BR [l]** | 51.8±19.4**^bc^** | 109.4±15.6**^ef^** | -1.6±13.3**^ac^** | 66.7±10.7**^df^** | 27.4±10.7**^ab^** | 89.7±7.1**^de^** |
| **Peak VE [l/min]** | 51.5±15.8**^bc^** | 51.8±13.1**^f^** | 44.4±10.1**^ac^** | 55.9±10.4**^f^** | 98.5±13.0**^ab^** | 90.1±6.1**^de^** |
| **Peak Vt [l/min]** | 1.3±0.3**^bc^** | 48.0±9.5**^f^** | 1.2±0.2**^ac^** | 46.5±8.0**^f^** | 2.6±0.2**^ab^** | 90.6±5.6**^de^** |
| **Peak Bf [1/min]** | 35.2±5.5**^bc^** | 69.9±10.0**^ef^** | 31.5±3.9**^ac^** | 63.0±7.7**^df^** | 47.6±5.2**^ab^** | 90.1±7.4**^de^** |
| **Peak VE/VO_2_** | 42.5±6.2**^c^** | 96.7±11.7**^ef^** | 40.1±6.0 | 89.2±10.1**^d^** | 39.5±4.7**^a^** | 91.8±8.4**^d^** |
| **Peak VE/VCO_2_** | 42.0±5.4**^bc^** | 125.1±16.9**^ef^** | 37.6±4.9**^ac^** | 108.0±14.1**^df^** | 28.4±2.7**^ab^** | 87.6±7.1**^de^** |
| **Peak PETO_2_ [mmHg]** | 113.8±4.8**^bc^** | 85.6±5.7**^ef^** | 108.3±5.7**^ac^** | 79.6±5.3**^df^** | 118.5±6.8**^ab^** | 90.8±7.2**^de^** |
| **Peak PETCO_2_ [mmHg]** | 34.8±3.7**^b^** | 92.0±9.6**^e^** | 38.6±5.1**^ac^** | 101.8±13.1**^df^** | 33.5±2.6**^b^** | 88.8±6.6**^e^** |
| **VAT % of pred. VO_2_/kg [%]** | 34.3±5.0**^bc^** | 60.9±8.9**^ef^** | 48.4±9.8**^ac^** | 80.1±9.1**^df^** | 60.2±9.2**^ab^** | 90.1±6.8**^de^** |
| **ECG grading [%]** | N/A | 79.6±12.2**^ef^** | N/A | 99.2±3.4**^df^** | N/A | 89.8±7.6**^de^** |
| **O_2_Pulse response grading [%]** | N/A | 74.4±12.2**^ef^** | N/A | 86.2±6.6**^df^** | N/A | 91.4±6.9**^de^** |
| **SaO_2_ [%]** | 97.8±1.5**^bc^** | 98.7±2.6**^ef^** | 88.5±4.9**^ac^** | 80.1±9.1**^df^** | 93.5±3.4**^ab^** | 89.0±7.0**^de^** |
| **VE/VCO_2_ slope** | 38.1±3.8**^bc^** | 130.3±11.3**^ef^** | 31.9±5.1**^ac^** | 105.6±16.6**^df^** | 25.4±2.4**^ab^** | 90.2±7.4**^de^** |
| **FVC [l]** | 3.6±0.8**^bc^** | 80.7±10.3**^ef^** | 2.5±0.5**^ac^** | 67.7±8.8**^df^** | 4.5±0.7**^ab^** | 90.2±7.5**^de^** |
| **FEV1 [l/sec]** | 2.8±0.6**^bc^** | 78.3±10.3**^ef^** | 1.2±0.2**^ac^** | 43.0±6.0**^df^** | 3.7±0.5**^ab^** | 90.7±6.3**^de^** |
| **FEV1/FVC [%]** | 88.2±8.8**^bc^** | 97.3±6.1**^ef^** | 49.0±4.1**^ac^** | 64.8±5.2**^df^** | 77.7±10.9**^ab^** | 89.1±7.3**^de^** |

**s-Table 3.** Comparisons of CPET results (actual and % of predicted**)** among the three studied groups – **The learning stage**

Data presented as mean ± SD.

WR **=** Work-Rate; VO_2_ = Oxygen Consumption; VCO_2_ = Carbon Dioxide Production; RER **=** Respiratory Exchange Ratio; HR = Heart Rate; O_2_Pulse = Oxygen Puls; BR = Breathing Reserve; VE = Minute Ventilation; Vt = Tidal Volume; Bf = Breathing Frequency; PETO_2_ = End-Tidal Oxygen tension; PETCO_2_ = End-Tidal Carbon Dioxide tension; VAT = Ventilatory Anaerobic Threshold; % predicted = percent of predicted normal value; ECG = Electrocardiography; SaO_2_ = Oxygen Saturation; FVC = [Forced Vital Capacity](http://medical-dictionary.thefreedictionary.com/forced+vital+capacity); FEV1 = Forced Expiratory Volume in 1 second; CHF = Chronic Heart Failure; COPD = Chronic Obstructive Pulmonary Disease; Healthy = Healthy normal participants.

^a^ Letters a, b, and c, represent significant differences (P < 0.05) related to measured values between the specified groups.

^b^ Letters d, e, and f, represent significant differences (P < 0.05) related to % of predicted values between the specified groups.

O_2_pulse response grading (% of predicted normal): Up-sloping 90%, flat 50%, down-sloping 30% [1, 2].

ECG (%) changes in ECG tracings were classified based on clinical severity: Normal 100%, nonspecific changes 80%, specific T-wave changes 75%, ventricular conduction defects 70%, atrial arrhythmia 60%, ST depression (>2 mm) 50%, ventricular arrhythmia 40%, ST elevation (>2 mm) 30%**.**
