# Supplementary material for: A Machine Learning Approach to the Interpretation of Cardiopulmonary Exercise Tests: Development and Validation
Source: Pulm Med. 2021 May 31;2021:5516248. doi: 10.1155/2021/5516248 (PMC8188599; doi:10.1155/2021/5516248)
Supplement: Supplementary 4 — s-Table 4. Comparisons of CPET results (actual and % of predicted) among the three studied groups—the validation stage. [file 5516248.f4.docx]

**s-Table 4.** Comparisons of CPET results (actual and % of predicted**)** among the three studied groups – **The validation stage**

| **Variables** | **CHF (n=23)** | | **COPD (n=25)** | | **Healthy (n=36)** | |
| --- | --- | --- | --- | --- | --- | --- |
|  | **Measured^a^** | **% of pred.^d^** | **Measured^b^** | **% of pred.^e^** | **Measured^c^** | **% of pred.^f^** |
| **Time [min]** | 8.1±1.8**^bc^** | 86.0±5.2**^ef^** | 12.2±2.1**^a^** | 101.5±14.8**^d^** | 12.2±2.7**^a^** | 104.2±18.2**^d^** |
| **Peak WR [watt]** | 74.7±38.1**^c^** | 40.9±16.2**^f^** | 57.6±21.1**^c^** | 43.7±18.5**^f^** | 175.7±84.1**^ab^** | 98.0±32.1**^de^** |
| **Peak VO_2_ [l/min]** | 1.0±0.3**^c^** | 40.5±10.5**^f^** | 0.9±0.2**^c^** | 53.4±14.8**^f^** | 2.3±1.1**^ab^** | 86.8±29.4**^de^** |
| **Peak VCO_2_ [l/min]** | 1.0±0.4**^c^** | 31.8±12.8**^f^** | 1.0±0.2**^c^** | 42.9±13.6**^f^** | 2.7±1.1**^ab^** | 80.5±24.0**^de^** |
| **RER** | 1.06±0.06**^c^** | 84.1±4.3**^f^** | 1.07±0.06**^c^** | 84.3±3.8**^f^** | 1.22±0.13**^ab^** | 94.2±10.1**^de^** |
| **Peak VO_2_/kg [ml/kg/min]** | 13.4±5.1**^c^** | 45.0±17.5**^ef^** | 18.1±5.9**^c^** | 68.5±19.3**^df^** | 41.6±14.2**^ab^** | 109.3±26.8**^de^** |
| **Slope VO_2_/WR** | 9.1±2.7**^c^** | 81.3±21.9**^f^** | 9.7±1.7 | 85.7±12.0 | 10.6±2.1**^a^** | 92.1±14.5**^d^** |
| **Peak HR [beat/min]** | 99.3±21.4**^bc^** | 49.0±9.6**^ef^** | 114.7±16.1**^ac^** | 59.9±8.6**^df^** | 164.0±27.0**^ab^** | 80.1±13.8**^de^** |
| **Peak O_2_Pulse [(ml/kg/beat)x100]** | 12.4±4.4**^c^** | 67.8±25.5**^f^** | 14.1±4.1**^c^** | 83.1±21.0**^f^** | 24.1±7.0**^ab^** | 114.1±28.0**^de^** |
| **BR [l]** | 42.2±18.4**^bc^** | 101.8±14.7**^ef^** | 17.3±11.1**^a^** | 81.8±8.9**^d^** | 24.6±13.1**^a^** | 87.7±10.5**^d^** |
| **Peak VE [l/min]** | 45.7±16.7**^c^** | 47.4±17.9**^f^** | 46.4±10.6**^c^** | 58.6±17.3**^f^** | 98.5±34.0**^ab^** | 93.5±22.6**^de^** |
| **Peak Vt [l/min]** | 1.3±0.5**^c^** | 51.1±32.6**^f^** | 1.3±0.3**^c^** | 51.7±11.0**^f^** | 1.9±0.7**^ab^** | 78.9±18.2**^de^** |
| **Peak Bf [1/min]** | 33.3±9.0**^c^** | 65.8±16.6**^f^** | 31.3±7.2**^c^** | 62.2±13.4**^f^** | 45.5±10.4**^ab^** | 87.2±17.1**^de^** |
| **Peak VE/VO_2_** | 48.7±12.2**^bc^** | 109.0±26.4**^ef^** | 40.5±7.2**^a^** | 89.2±13.4**^d^** | 40.8±7.4**^a^** | 94.1±15.3**^d^** |
| **Peak VE/VCO_2_** | 46.5±10.2**^bc^** | 136.5±30.0**^ef^** | 39.8±7.3**^ac^** | 112.8±19.6**^d^** | 33.6±5.3**^ab^** | 101.1±14.7**^d^** |
| **Peak PETO_2_ [mmHg]** | 114.1±5.6**^c^** | 85.9±6.7 | 110.9±5.3 | 82.3±6.1**^f^** | 114.6±7.3**^a^** | 87.0±8.3**^e^** |
| **Peak PETCO_2_ [mmHg]** | 33.8±4.8 | 89.6±12.3 | 35.5±4.3 | 93.9±11.1 | 36.7±5.3 | 96.8±13.7 |
| **VAT % of pred. VO_2_/kg [%]** | 33.3±11.9**^bc^** | 57.3±17.0**^ef^** | 49.5±13.7**^ac^** | 79.9±14.9**^df^** | 59.0±12.2**^ab^** | 89.3±10.2**^de^** |
| **ECG grading [%]** | N/A | 79.6±19.0**^ef^** | N/A | 97.9±6.1**^d^** | N/A | 96.7±13.9**^d^** |
| **O_2_Pulse response** **grading [%]** | N/A | 71.3±21.9 | N/A | 77.6±19.8 | N/A | 81.7±19.9 |
| **SaO_2_ [%]** | 97.8±1.6**^bc^** | 98.7±3.1**^ef^** | 92.9±3.8**^ac^** | 88.3±7.7**^df^** | 95.7±1.8**^ab^** | 94.3±4.5**^de^** |
| **Slope VE/VCO_2_** | 46.2±17.6**^bc^** | 156.2±57.3**^ef^** | 38.0±11.3**^ac^** | 124.4±36.9**^d^** | 30.3±6.5**^ab^** | 109.9±23.9**^d^** |
| **FVC [l]** | 4.0±1.4 | 84.3±19.4 | 3.4±1.0 | 87.5±22.9 | 3.9±0.9 | 83.7±12.0 |
| **FEV1 [l/sec]** | 3.0±1.3**^b^** | 81.4±24.5**^e^** | 1.4±0.5**^ac^** | 53.0±19.7**^df^** | 3.1±0.9**^b^** | 80.3±12.3**^e^** |
| **FEV1/FVC [%]** | 86.2±19.6**^b^** | 96.7±18.8**^e^** | 54.5±16.7**^ac^** | 70.3±17.6**^df^** | 88.9±15.0**^b^** | 96.8±11.0**^e^** |

Data presented as mean ± SD.

See e-Table 2 for expansion abbreviations.

^a^ Letters a, b, and c, represent significant differences (P < 0.05) related to measured values between the specified groups.

^b^ Letters d, e, and f, represent significant differences (P < 0.05) related to % of predicted values between the specified groups.

O_2_pulse response grading (% of normal): Up-sloping 90%, flat 50%, down-sloping 30% [1, 2].

ECG grading (% of normal), changes in ECG tracings were classified based on clinical severity: Normal 100%, nonspecific changes 80%, specific T-wave changes 75%, ventricular conduction defects 70%, atrial arrhythmia 60%, ST depression (>2 mm) 50%, ventricular arrhythmia 40%, ST elevation (>2 mm) 30%**.**
